# Emergencies in Hematology: Why, When and How I Treat?

**DOI:** 10.3390/jcm13247572

**Published:** 2024-12-12

**Authors:** Andrea Duminuco, Vittorio Del Fabro, Paola De Luca, Dario Leotta, Miriana Carmela Limoli, Ermelinda Longo, Antonella Nardo, Gabriella Santuccio, Alessandro Petronaci, Gaia Stanzione, Francesco Di Raimondo, Giuseppe Alberto Palumbo

**Affiliations:** 1 Hematology Unit with BMT, A.O.U. Policlinico “G.Rodolico-San Marco”, 95123 Catania, Italy; paodeluca37@gmail.com (P.D.L.); dario.leotta96@gmail.com (D.L.); mirianalimoli@virgilio.it (M.C.L.); lindalongo96@gmail.com (E.L.); antonella.nardo5@gmail.com (A.N.); santuccio.gabriella@gmail.com (G.S.); alessandropetronaci@outlook.it (A.P.); gaiastanzione97@gmail.com (G.S.); francesco.diraimondo@unict.it (F.D.R.); palumbo.gam@gmail.com (G.A.P.); 2 Faculty of Medicine and Surgery, “Kore” University of Enna, 94100 Enna, Italy; vdelfabro@yahoo.it

**Keywords:** emergencies, septic shock, TLS, hyperleukocytosis, hypercalcemia, DIC, transfusion reactions, CAR-T complications, PTT, sickle crisis

## Abstract

Hematological emergencies are critical medical conditions that require immediate attention due to their rapid progression and life-threatening nature. As various examples, hypercalcemia, often associated with cancers such as multiple myeloma, can lead to severe neurological and cardiac dysfunction. Hyperleukocytosis, common in acute myeloid leukemias, increases the risk of leukostasis and multiorgan failure. Sickle cell crisis, a common complication in sickle cell disease, results from vaso-occlusion, leading to acute pain and tissue ischemia. Tumor lysis syndrome, reported in cases of rapid destruction of cancer cells, causes electrolyte imbalances and acute kidney injury. Acute transfusion reactions, fundamental in hematological conditions, can range from mild allergic responses to severe hemolysis and shock, requiring prompt management. Disseminated intravascular coagulation, involving excessive coagulation and bleeding, is commonly triggered by hematological malignancies, common in the first phases of acute promyelocytic leukemia. Recently, in the era of bispecific antibodies and chimeric antigen receptor T cells, cytokine release syndrome is a manifestation that must be recognized and promptly treated. Understanding the pathophysiology, recognizing the clinical manifestations, and ensuring adequate diagnostic strategies and management approaches for each condition are central to early intervention in improving patient outcomes and reducing mortality.

## 1. Introduction

Hematological emergencies encompass a range of acute, life-threatening conditions involving the blood, its components, or hematopoietic organs. These conditions demand immediate recognition and intervention. The dynamic and often unpredictable nature of, for example, hypercalcemia in multiple myeloma, hyperleukocytosis in acute leukemias, and disseminated intravascular coagulation common in promyelocytic leukemia, means that delayed diagnosis or treatment can result in rapid clinical deterioration and death. Prompt identification of the underlying pathology and timely, targeted interventions are essential to improving patient outcomes and reducing mortality. Early diagnosis and swift therapeutic measures with adequate management are key in achieving optimal patient recovery.

## 2. Hypercalcemia

Hypercalcemia is defined as an increase in the serum calcium level above the upper limit of normal. Hypercalcemia of malignancy is a metabolic emergency occurring in advanced solid neoplasms (breast, lung, renal cancers), as well as in hematological disorders such as multiple myeloma, high-grade lymphoma, and acute or chronic leukemias [1]. Based on serum calcium levels, hypercalcemia can be mild (10.5–11.9 mg/dL), moderate (12–13.9 mg/dL), or severe (>14 mg/dL). To calculate the calcium level, it is necessary to consider that, in hypo-/hyperalbuminemia, it must be corrected, since 40% of serum calcium is bound to albumin, as detailed below.

- Albumin < 40 g/dL: corrected calcium = (Ca^2+^) + 0.02 [40 − (albumin)]
- Albumin ≥ 40 g/dL: corrected calcium = (Ca^2+^) − 0.02 [(albumin) − 45]

### 2.1. Symptoms

The gravity of clinical symptoms depends on the severity of the electrolytic disorder: the mild form is usually asymptomatic but can also cause fatigue and constipation in about 20% of cases and is generally associated with constitutional symptoms such as weakness or lassitude [2]. In severe cases, mental changes may occur, along with a slowing of the central, peripheral, and autonomic nervous systems, personality changes, and, ultimately, coma [3]. Other symptoms can affect several organs: gastrointestinal tract (anorexia, nausea, vomiting, abdominal pain up to peptic ulceration and pancreatitis as rare complications), genitourinary system (polyuria, polydipsia), cardiovascular system (QT interval on ECG, T-wave flattening or inversion, ST-elevation, presence of J-wave at the end of the QRS complex, cardiac arrhythmias and hypertension) and kidneys (dehydration, which could cause acute renal failure and renal calculi) [4].

If not promptly treated, it is related to a higher risk of mortality and can lead to organ failure and sepsis. Severe hypercalcemia in an uncontrolled malignancy at hospital admission is associated with a higher mortality rate compared to patients who have normal or low calcium blood levels; in fact, it could be a marker of the advanced stage of neoplastic disease [5]. The overall survival in these cases at 30 days is around 50% of patients, and up to 75% die within three months of diagnosis [6].

### 2.2. Pathophysiology

Different explanations of hypercalcemia in hematological malignancies have been formulated. In multiple myeloma, osteoclast activation stimulates bone resorption through receptor activator of nuclear factor Κ B-ligand (RANKL), macrophage inflammatory proteins (MIP1α), IL-6, and tumor necrosis factor-α (TNF-α) pathways, with increased calcium release into the circulation [7]. Moreover, several studies also showed the capacity of plasma cells to produce parathyroid hormone-related protein (PTHrP), which regulates their survival and pro-osteoclastic activity [8]. Almost 13% of patients affected by multiple myeloma show hypercalcemia at diagnosis [9].

Hypercalcemia is also observed in more than 80% of adult T-cell leukemia/lymphoma (ATLL) patients at diagnosis, and a significant role in its presentation may be played by a high serum MIP-1α level, spontaneously produced by ATLL cells, which induces the expression of RANKL on these cells and the differentiation of monocytes into osteoclasts [10].

Hypercalcemia may also be present in Hodgkin’s and non-Hodgkin’s lymphomas [11]. Hewison and colleagues suggested that it may be caused by the dysregulated overproduction of calcitriol by the tumor microenvironment cells, such as spleen macrophages [12]. Other mechanisms involved in the genesis of hypercalcemia may be the production of interleukins and prostaglandins (such as E2), as seen in chronic myeloid leukemia [13], an ectopic activity of 1-α-hydroxylase leading to the formation of 1,25 dihydroxycholecalciferol and ectopic production of parathyroid hormone (PTH) [6].

### 2.3. Management

Patients with asymptomatic or mild symptomatic hypercalcemia (<12 mg/dL) do not require aggressive treatment [6].

In cases of symptoms, prompt therapy is mandatory. It requires simultaneous administration of intravenous normal saline, typically 1–2 L in the first hour followed by 2 L at 200 mL/hour with close monitoring of volume status, and intravenous bisphosphonate in patients without renal impairment (disodium pamidronate 60–90 mg IV over 2 h, or zoledronic acid 4 mg IV in 100 mL normal saline over at least 15 min) that allows a more sustained control of the hypercalcemia (for 2 to 4 weeks) [14]. Subcutaneous calcitonin (200 IU every 8 h) can be administered while waiting for the peak of bisphosphonate action [6].

Denosumab (a monoclonal antibody inhibitor of osteoclast activity) is alternatively administered in bisphosphonate-refractory cases and patients with kidney failure because of its non-renal clearance [6]. It prevents bone resorption by targeting RANK ligands, therefore inhibiting osteoclastic activity. Denosumab proved its efficacy in preventing excessive bone loss both in metastatic and early cancer-diagnosed patients and also showed efficacy in reducing prostate cancer progression [15].

Other possible treatments are gallium nitrate, cinacalcet (preferentially used in hemodialysis patients and parathyroid cancer), plicamycin, and corticosteroids (e.g., oral prednisolone 60 mg/daily that can be increased up to 25 mcg/kg, administered intravenously three times weekly), usually effective when hypercalcemia is the consequence of excessive intestinal calcium absorption (vitamin D intoxication, granulomatous disorders, lymphomas) [2].

Also, lithium carbonate, volume depletion, prolonged inactivity, high-calcium diet (>1000 mg/day), and calcium/vitamin D supplements must be excluded since they can exacerbate hypercalcemia and kidney injury in inadequately hydrated patients. Loop diuretics must be used carefully and given only after adequate intravenous resuscitation [14].

Hemodialysis is the treatment of choice in patients with heart failure or renal insufficiency. To prevent a recurrence, the underlying malignancy must be treated, and follow-up therapy is recommended. Recovery usually requires at least 3 to 5 days [4].

A brief therapeutic summary is reported in Table 1.

## 3. Hyperleukocytosis and Leukostasis

Hyperleukocytosis (HL) is a hematological condition defined by a white blood cell (WBC) count >50 × 10^9^/mm^3^ in the peripheral blood of hematological patients, not related to any infection [16].

Leukocytes higher than 100 × 10^9^/mm^3^ are often associated with life-threatening events, such as leukostasis, defined as a reduction in blood flow due to hyperviscosity, disseminated intravascular coagulation (DIC), and tumor lysis syndrome (TLS), representing hematological emergencies that require prompt treatment with a cytoreductive strategy due to the high related mortality [17].

Hyperleukocytosis is observed chiefly with an incidence range of 10~30% in newly diagnosed acute lymphoblastic leukemia (ALL) and acute myeloid leukemia (AML). It has also been reported (with lower incidence) in chronic conditions, such as myeloid leukemia (CML) and myeloproliferative neoplasms (MPN), and this variable is considered in the commonly used prognostic scores [18,19].

### 3.1. Pathophysiology

The volume of leukocytes and their poor deformability are directly responsible for hyperviscosity syndrome and occlusive clinical manifestations. In particular, the lower deformability that characterizes myeloid blasts is the reason for the higher incidence of symptomatic hyperleukocytosis in AML [20].

Conversely, the mechanism at the origin of leukostasis is poorly understood. Among the causes responsible for the mobilization of WBC from bone marrow to blood flow, the pro-inflammatory environment associated with acute leukemia is responsible for increased production of cytokines such as TNF-α and IL-1β that are directly involved in cell adhesion; moreover, blast cells that progressively accumulate on inactive endothelium induce endothelial cell adhesion receptor expression (E-selectin, P-selectin, VCAM-1, and ICAM-1), promoting their own adhesion and tissue invasion [21]. Another factor is the expression of surface markers such as cluster of differentiation 11c (CD11c) on leukemic blasts.

Recent studies underlined how the expression of specific surface markers significantly impacts leukostasis severity. Notably, the expression of CD11c (mostly shown on the surface of leukemic monocytic and myelomonocytic blasts) allows the interaction with ICAM molecules and results in the development of a more severe phenotype of HL [22].

Hyperleukocytosis has a high morbidity index. Mortality rates as high as 8% and 29% within 24-h and one-week periods, respectively, have been reported [23]. To date, the presence of FMS-like tyrosine kinase 3-internal tandem duplication (FLT3-ITD) mutations has been associated with a higher incidence of hyperleukocytosis. Still, there are not enough studies to correlate the molecular biology of AML and HL, so the role of FLT3 mutations and their therapeutic implication remain ambiguous [24,25]. 

### 3.2. Signs and Symptoms

According to the significant early mortality risk in hyperleukocytic leukemia, Novotny and colleagues proposed a score predicting the likelihood of leukostasis based on the various clinical features due to occlusive disease of the microvasculature (Table 2) [26].

Increased leukocytes and upregulation of several adhesion molecules lead to reduced blood flow, leading to blast recruitment and wall disruption with a perivascular tissue infiltration directly responsible for the hypoxic damage. The probability of early mortality is strictly linked to pulmonary signs and neurological symptoms [27].

Pulmonary signs like tachypnea, dyspnea, shortness of breath, and cough are studied through computerized tomography (CT), while chest X-ray may be expected [28]. Central nervous system (CNS) manifestations of leukostasis, like confusion, blurred vision, and focal neurological deficits, are better studied through CT and magnetic resonance imaging (MRI), which are able to reveal ischemic areas and/or hemorrhages. A fundoscopic exam is also helpful in establishing papilledema, retinal bleeding, and a red flag of intracranial hypertension [20]. Other potential clinical manifestations, such as congestive heart failure, priapism, acral lividosis, avascular bone necrosis, acute kidney injury, retinopathy, acute appendicitis, and deep venous thrombosis, must be taken into consideration (Table 3).

### 3.3. Management

A recent systematic review found no evidence of standardized guidelines for managing hyperleukocytosis. Clinical choices are crucial within the first hours, considering that managing hyperleukocytosis remains a medical challenge. The treatment rationale is based on cytoreduction, which can be achieved by mechanical procedures, like leukapheresis, or pharmacologic strategies to prevent adverse events related to increased viscosity.

A milestone in the management of hyperleukocytosis is cytoreductive therapy through intensive chemotherapy regimens (low-dose cytarabine and hydroxyurea). At the same time, the role of leukapheresis remains controversial [29,30]. Leukapheresis is a type of apheresis procedure used to withdraw leukocytes from peripheral blood. This approach allows rapid reduction of the number of leukocytes. To perform leukapheresis, special cell separators collect white blood cells from peripheral blood, working with blood flow set on the patient’s features (sex, weight, height, hematocrit, and pre-apheresis WBC count). A single cycle can reduce WBC by 30–60% [18].

Leukapheresis has been demonstrated to play a direct role as a cell cycle modulator. It can stop tumor cells in the S phase and enhance tumor cell sensitivity to cytarabine [30].

Although leukapheresis is a safe and efficient procedure, it is an invasive technique with different side effects (hypocalcemia, bleeding risk, allergic reaction) [18].

Nowadays, leukapheresis is considered a second-line therapy (Category II recommendation—Grade 2B) for patients with symptomatic hyperleukocytosis, while its use remains controversial in asymptomatic patients (Category III—Grade 2C) [31].

The employment of leukapheresis in acute promyelocytic leukemia (APL) is still problematic. Although most recent studies suggest the safe and beneficial effect of this procedure or at least a non-significant difference in overall survival among patients undergoing leukapheresis and those not, the role of leukapheresis remains unclear in managing APL [32]. APL is a subtype of acute myeloid leukemia (older M3 FAB) with a very high incidence of pre-treatment mortality. The t(15;17) (q22;q21), occurring in the majority of APL cases, determines PML::RARA rearrangement, which encodes for the PML/RAR alpha fusion protein able to stop myeloid differentiation at the promyelocytic stage [33]. The increased amount of promyelocytes expressing tissue factor (TF) on their surface is the direct mechanism responsible for a procoagulant state [32]. According to colleagues, the employment of leukapheresis not only exacerbates the coagulopathy [34], but could lead to fatal or near-fatal events, primarily due to bleeding [35].

Cytoreduction by pharmacological regimens has a central role in asymptomatic HL. In AML, hydroxyurea (HU), administered as pre-treatment at the dose of 50–60 mg/kg per day, resulted in an efficient emergency strategy to reduce the rate of early death in patients with HL onset before starting induction chemotherapy [36]. Moreover, HU can be employed in patients with several comorbidities, like renal insufficiency and electrolyte disturbances, deemed to be “unfit” for intensive chemotherapy regimens [37].

## 4. Tumor Lysis Syndrome

Tumor lysis syndrome (TLS) is an oncologic emergency due to a massive cellular break induced by cytotoxic therapy, which may evolve until acute kidney injury and severe arrhythmias. The incidence of TLS is variable according to the type of cancer, with a stronger association with hematologic malignancies [38].

It may be spontaneous in high-rate proliferation neoplasms (such as acute myeloid leukemia or high-grade lymphomas presenting with hyperleukocytosis of bulky disease), or therapy-related.

It is divided into laboratory and clinical forms. The metabolic panel monitors common TLS-related metabolic imbalances such as hyperkalemia, hypocalcemia, hyperphosphatemia, and hyperuricemia. Blood urea nitrogen, creatinine, and lactate dehydrogenase levels should be monitored 2–3 times daily to track TLS onset.

The first form is defined by the presence of at least two of the following laboratory alterations (Cairo and Bishop criteria) [39]:

- uric acid ≥ 476 mmol/L or 25% increase from baseline;
- potassium ≥ 6 mmol/L or 25% increase from baseline;
- calcium ≤ 1.75 mmol/L or 25% decrease from baseline;
- phosphate ≥ 1.45 mmol/L or 25% increase from baseline.

The clinical form is diagnosed by the presence of laboratory alterations associated with clinical symptoms, such as gastrointestinal (nausea, vomiting, diarrhea), neurological symptoms (lethargy, myalgia, muscle cramps, paresthesia, tetany, seizures), and cardiological symptoms (cardiac arrhythmias, syncope), until death [40].

Conditions most commonly associated with tumor lysis syndrome include clinically aggressive non-Hodgkin lymphomas, acute lymphoblastic leukemia, acute myeloid leukemia, and Burkitt lymphoma, above all for the high proliferative rate, and demonstrate significant sensitivity to chemotherapy [41]. Specifically, acute myeloid or lymphoblastic leukemias with a WBC count of 100,000/µL or more, Burkitt lymphoma, or diffuse large B-cell lymphoma with elevated lactate dehydrogenase or bulky disease (>10 cm) are neoplastic diseases with high risk of TLS [42].

Several reports of TLS occurring spontaneously before the initiation of chemotherapy have been documented in patients with non-Hodgkin lymphomas, acute leukemias, and breast cancer [43]. 

Beyond the neoplasm’s features, certain patient factors can also predispose to an increased risk of TLS. Patients at higher risk for TLS include those with pretreatment hyperuricemia, preexisting renal issues, oliguria, and volume depletion, associated with older age and the use of concomitant drugs that increase uric acid levels. Impaired renal function significantly increases tumor lysis syndrome risk, necessitating close monitoring and managing fluid status and uric acid levels before and during treatment [44].

The exact incidence of TLS is not adequately assessed and remains unclear, though studies provide some insight. In a retrospective analysis of 102 patients with high-grade non-Hodgkin lymphoma, TLS occurred in 42% of cases, but only 6% were clinically significant, without correlation with ethnicity and sex [45].

In another study involving 951 newly diagnosed cancer patients, laboratory TLS occurred in 9.3%, and clinical in 6.7%. Among the hematological conditions, acute leukemia and multiple myeloma showed the highest incidence rates. Laboratory TLS was primarily marked by hyperuricemia, while clinical TLS was associated with renal insufficiency and cardiac arrhythmias [46].

Finally, a study of 788 adult and pediatric patients with acute leukemia or non-Hodgkin lymphoma across different European centers showed an incidence of 18.9% for laboratory TLS and 5% for clinical. Acute lymphoblastic leukemia, more common in pediatric patients, had the highest rates of both laboratory (21.4%) and clinical manifestations (5.2%), followed by non-Hodgkin lymphoma and acute myeloid leukemia [47].

### 4.1. Symptoms and Management

The clinical TLS form is characterized by one or more of the following symptoms, caused by electrolytic alterations.

The first clinical signs appear in the first 48–72 h after chemotherapy starts, but laboratory data may be present already in the first 6–24 h [38].

Imaging, electrocardiography (ECG), and laboratory assessments are essential in evaluating and managing TLS [38].

Chest X-rays and CT scans are used to assess mediastinal masses and pleural effusions and to evaluate potential kidney damage. ECG is vital for detecting cardiac abnormalities caused by hyperkalemia and hypocalcemia, especially since hyperkalemia can lead to life-threatening arrhythmias.

The metabolic panel monitors common TLS-related metabolic imbalances such as hyperkalemia, hypocalcemia, hyperphosphatemia, and hyperuricemia. Blood urea nitrogen, creatinine, and lactate dehydrogenase levels should be checked 2–3 times daily to track TLS onset.

The management of TLS starts with a targeted prevention, consisting of the following:

- High grade of intravenous hydration, starting at least 24 h before the therapy begins, with the aim of 3 L/day of diuresis or >100 mL/h;
- Hypouricemic therapy, with allopurinol (in low-medium risk patients, up to 600–800 mg/day, starting 2–3 days before treatment and continuing for about 10–14 days) or rasburicase (in high-risk patients, at 0.2 mg/kg intravenous for 3–7 days, depending on clinical and biochemical data). Rasburicase is a highly potent uricolytic agent that catalyzes the enzymatic oxidation of uric acid into allantoin, a water-soluble product that is easily excreted by the kidneys in the urine. A randomized phase 3 comparative study utilizing the recommended dose demonstrated a significantly faster onset of action for rasburicase than allopurinol. Four hours after the first dose, there was a significant difference (*p* < 0.0001) in the mean percentage change from baseline plasma uric acid levels between the rasburicase group (−86.0%) and the allopurinol group (−12.1%) [48]. Furthermore, febuxostat seems to be a valid alternative to allopurinol in patients with compromised kidney function, allopurinol intolerance, or resistance, where rasburicase is not available [38,49].

#### 4.1.1. Hyperuricemia

DNA is made up of smaller units called nucleotides, which serve as the molecule’s building blocks. Each nucleotide has three key components: a phosphate group, a sugar group, and a nitrogenous base (adenine, thymine, guanine, or cytosine). Adenine and guanine are classified as purines, while thymine and cytosine are pyrimidines. In RNA, the sugar is ribose, and uracil replaces thymine as one of the nitrogenous bases.

The metabolism of adenine and guanine (nitrogenous bases in the DNA) occurs stepwise. Adenine is converted to hypoxanthine and guanine to xanthine, metabolized into uric acid by the enzyme xanthine oxidase. In most mammals (not in humans), uric acid is broken down by urate oxidase into allantoin (the aforementioned role of rasburicase), a more water-soluble compound easily excreted by the kidneys. 

In TLS, excessive uric acid can crystallize in the renal tubules, leading to obstructive uropathy, reduced glomerular filtration rate, vasoconstriction, and kidney ischemia. Additionally, uric acid acts as a pro-inflammatory agent, promoting the release of cytokines like TNF-α, worsening kidney damage [45,50]. Reducing the amount of circulating uric acid is crucial to avoid serious complications.

#### 4.1.2. Hyperphosphatemia

Caused by phosphate release from the nucleic acids of lysed tumor cells that contain a phosphorus concentration up to four times higher than in normal cells, calcium-phosphorus salt deposits can precipitate in the kidneys and cardiac tissues, potentially causing cardiac arrhythmias [51]. Urinary alkalization is not indicated because of the risk of calcium-phosphate precipitation in the renal tubules, and it can cause xanthine nephropathy by decreasing the solubility of xanthine [44]. Therapy consists of reducing phosphate intakes and using noncalcium-phosphate bindings [38].

#### 4.1.3. Hyperkalemia

Intracellular potassium concentrations are approximately 120 to 130 meq/L, and the rapid lysis of neoplastic cells releases this electrolyte into the bloodstream, with a higher risk of arrhythmia and uric acid obstructive uropathy. Continuous monitoring of cardiac rhythm and a neurologic consultancy are recommended. Mild forms can be treated with sodium polystyrene sulfonate [44], while severe hyperkalemia therapy consists of intravenous infusion of 10% dextrose and rapid-acting insulin to promote a K^+^ cellular uptake. Another option is oral gastrointestinal sodium-potassium exchange resins [38].

#### 4.1.4. Hypocalcemia

Due to phosphorus chelation, the asymptomatic forms generally resolve with phosphate correction; symptomatic forms can be treated with calcium supplementation, avoiding potential complications, such as arrhythmias, tetany, seizures, and death [52].

#### 4.1.5. Renal Replacement Therapy

The choice depends on laboratory data, cellular turnover rate, and the patient’s general clinical condition. Intermittent hemodialysis can be used in patients with hyperkalemia and hyperuricemia, while continuous hemodialysis is used in critical situations. The mandatory indications for hemodialysis include severe oliguria or anuria, fluid overload, persistent hyperkalemia, symptomatic hypocalcemia secondary to hyperphosphatemia, and a calcium-phosphate product of ≥70 mg²/dL [53].

## 5. Neutropenic Fever and Septic Shock

Neutropenic fever (NF) and septic shock (SS) are critical complications in hematological conditions, representing emergencies with considerable morbidity and mortality. NF occurs in up to 80% of patients with hematologic malignancies, with mortality rates ranging from 5% to 20%, and potentially reaching 50% if SS ensues [54]. NF is most prevalent before engraftment during hematopoietic cell transplantation and following induction chemotherapy for acute leukemias. It is defined by a corporal temperature exceeding 38.3 °C or a persistent temperature over 38.0 °C for more than one hour, accompanied by an absolute neutrophil count (ANC) below 500 cells/mm³, or a rapid decline to this threshold within 48 h [55].

The infection risk significantly increases when the neutrophils fall below 100 cells/μL, particularly in individuals with prolonged neutropenia (>7 days).

The pathophysiology of NF involves alterations in hematopoiesis, resulting in quantitative and qualitative neutrophil deficiencies and damage to mucosal barriers, facilitating bacterial translocation. Specific hematological disorders, including abnormal antibody production (as reported also for SARS-CoV-2 vaccinations), T-lymphocyte deficiency, and splenectomy, further impair the host’s immune defense, increasing susceptibility to bacterial, fungal, and viral infections [56,57,58,59,60]. SS is characterized by a dysregulated host immune response to microbial invasion, triggering a cytokine storm and systemic inflammation, which can lead to widespread vasodilation, capillary leakage, and multiorgan dysfunction [61].

### 5.1. Symptoms, Diagnosis, and Prognosis

Patients with NF may lack the typical inflammatory responses observed in localized infections, often presenting solely with fever. The use of glucocorticoids can obscure fever and complicate diagnostic efforts [55].

SS clinically manifests with confusion and attention deficits; initially, warm skin transitions to a mottled appearance with cold, pale extremities. Severe hypotension (mean arterial pressure < 65 mmHg), reduced urine output (<0.5 mL/kg/min), and desaturation may culminate in multiorgan failure and mortality [55].

The Multinational Association for Supportive Care in Cancer (MASCC) score is commonly used for risk stratification. It considers various factors, including the severity of NF based on disease burden, symptomatology, age, comorbidities, and ambulatory status. Low-risk patients (MASCC score > 21) may be candidates for outpatient oral empirical therapy. High-risk patients (MASCC score < 21) should be hospitalized and receive intravenous antibiotic therapy due to their high likelihood of severe complications [55].

Initial diagnostic tests should include a complete blood count with differential leukocyte count, a comprehensive metabolic panel, two sets of blood cultures (one from each lumen of a central venous catheter and one from a peripheral venous site), and serum inflammatory markers. Cultures from various sites (urine, stool, skin, cerebrospinal fluid, respiratory samples) should be performed based on clinical presentation. New technologies, such as polymerase chain reaction panels and other rapid diagnostic tests, improve the ability to identify a pathogen and reduce identification time. If respiratory symptoms are present, a chest X-ray should be performed, followed by a chest CT or other appropriate imaging, if necessary. In cases of SS, arterial blood gas analysis, including lactate measurement, is crucial [55,62].

In patients with NF, the infection often remains microbiologically undiagnosed. According to a study by Choi Wan Chan et al., only 40.1% of episodes had a documented origin. Bacterial infections accounted for 20.2% of cases, with Gram-negative bacteria (11.8%) being predominant. The most commonly encountered pathogen was Escherichia coli (7%), representing a significant proportion of infections. Among Gram-positive bacteria (9.9%), methicillin-resistant Staphylococcus aureus (5.1%) was the most prevalent. Microbiologically diagnosed fungal infections constituted 19.9% of cases, with Candida albicans being the most common (1.6%), followed by other pathogens. When the site of infection was identified, the gastrointestinal tract was the most frequent, followed by the lower respiratory tract, head and neck, skin and soft tissue, and catheter insertion sites. The highest culture positivity rates were observed in blood cultures, followed by sputum and pus [63,64]. Other pathogens could be opportunistic agents, emerging fungi, and parasites [65,66].

### 5.2. Management

Current guidelines recommend initiating empiric broad-spectrum antibiotics within the first hour of NF presentation. Initial therapy typically involves monotherapy with an anti-pseudomonal β-lactam agent, such as cefepime, piperacillin-tazobactam, or carbapenems, which cover a broad range of pathogens. Aminoglycosides, fluoroquinolones, and vancomycin can be added if resistance is suspected or complications arise. Empirical antifungal coverage should be included if the patient remains febrile or hemodynamically unstable after 4–7 days with negative blood cultures. The antimicrobial regimen should include linezolid, teicoplanin, daptomycin, and tigecycline. Antimicrobials for Gram-positive microorganisms are used empirically only in specific situations and should be discontinued 48 h after initiation if not confirmed [55]. For SS, initial management includes fluid infusion (30 mL/kg) within the first hour and is completed within 3 h. If hypotension persists, vasopressor therapy, typically with norepinephrine at 0.1–1.2 μg/kg/min, is initiated. Additional therapies for refractory shock may include glucocorticoids, inotropic support, and blood transfusions. Based on oxygen saturation levels, supportive oxygen therapy and potentially mechanical ventilation should be considered [61]. Association between different fungal drugs appears to confer better results, above all for emerging infections [67].

NF and SS are life-threatening complications in patients with hematologic diseases undergoing chemotherapy. Early recognition and timely initiation of appropriate empiric therapy are critical for improving patient survival. Risk stratification using tools such as the MASCC score can help determine the level of care and the type of treatment needed. At the same time, comprehensive diagnostic assessments are essential for guiding specific therapies. A multidisciplinary approach involving hematologists, infectious disease specialists, and intensivists is crucial for improving clinical outcomes and reducing mortality associated with these hematologic emergencies.

## 6. Blood Transfusion Reactions

Blood transfusions are a form of supportive therapy commonly employed in clinical practice, especially in the hematologic field, considering the predisposition to develop anemia and/or thrombocytopenia due to defective or insufficient production and/or accelerated peripheral destruction of red blood cells, hemoglobin, and platelets, as well as the therapies’ myelotoxic side effects.

Although blood component transfusions are a relatively safe practice, they can be associated with undesirable effects due to immunological or other causes. These can be acute or delayed, depending on whether their onset occurs immediately following transfusion initiation, within 24 h from the unit infusion responsible for the reaction, or in the following days.

For example, in Italy, in 2017, an incidence of 2041 undesirable effects among patients who had received blood components was reported, accounting for one reaction every 1450 transfusions. However, severe reactions requiring resuscitation procedures or resulting in patients’ death were one in every 134,575 transfused units.

### Subtypes and Management

Not every blood transfusion reaction constitutes a hematologic emergency. On the contrary, most of them comprise mild and transient reactions that are self-limiting or can be easily managed by stopping the transfusion and with antihistamine and steroid therapy. Severe reactions, on the other hand, must be quickly identified and treated to reduce morbidity and mortality related to them. Such conditions include acute hemolytic anemia due to immunological incompatibility, transfusion-related acute lung injury (TRALI), transfusion-associated circulatory overload (TACO), and transfusion-related sepsis (Table 4).

Acute hemolytic transfusions (AHT) primarily result from red blood cell transfusion, even though they can occur with any blood component. AHT is the result of the incompatibility of the blood system. In most instances, this phenomenon is related to AB0 system incompatibility, but hemolytic reactions could result from the involvement of other antigens, such as Kell and Duffy. The first scenario is also the most severe in terms of clinical impact on the recipient. The pathogenetic events leading to transfused blood cell lysis depend on the presence of antibodies, which can either activate the complement system, producing intravascular hemolysis, or bind the erythrocyte’s surface and cause extravascular hemolysis through the intervention of macrophages. Life-threatening AHT can manifest with hypotension, diffuse myalgias, dyspnea, airway closing, and fever. Laboratory features include a positive Coombs test, an increase in lactate dehydrogenase, indirect bilirubin, reduced haptoglobin, and hemoglobinuria. In such instances, the transfusion must be stopped, and the patients should be treated with steroids, antihistamines, and with epinephrine in case of airway closing [68].

TACO is a life-threatening condition whose onset occurs during or within 12 h from the beginning of a blood component transfusion. Patients usually develop hypotension, acute respiratory distress, tachypnea, tachycardia, cough, cyanosis, headache, positive water balance, pulmonary edema, and fever. TACO’s pathogenesis is still unknown, compromising the chance of developing specific therapies.

TRALI is a complex and severe condition characterized by acute lung injury and respiratory distress occurring usually 6 h after a blood component transfusion. One of the most accepted hypotheses attributes a significant pathogenetic role to neutrophils, which can cause endothelial damage through reactive oxygen species (ROS) production, resulting in non-cardiogenic edema [69]. Patients with TRALI usually show fever, hypotension, tachypnea, cyanosis, and worsening dyspnea.

As of now, there are no specific therapeutic options for both TACO and TRALI, which means their management entirely depends on supportive measures. Supportive measures for TACO comprise oxygen therapy, intubation, and diuresis. TRALI’s management includes oxygen, intubation, and scrupulous evaluation of hemodynamic parameters. Promising future TRALI treatment programs may include interleukin 10 (IL-10) therapy, anti-IL8 therapies, or therapies blocking ROS [70].

Transfusion-related sepsis is caused by infusion in the bloodstream of bacteria capable of surviving at low temperatures. It is more commonly associated with the transfusion of platelet units stored at room temperature, while it is rarely the consequence of transfusing red blood cells stored at refrigerator temperature. Patients may have fever, rigors, abdominal pain, vomiting, diarrhea, and severe hypotension [71]. The clinical evaluation and the following management do not differ from those employed in case of septic scenarios of any other etiology, and for the discussion of this topic, refer to the dedicated paragraph.

Regardless of the measures required based on the specific clinical setting, stopping the transfusion whenever a reaction is suspected while keeping an intravenous line open with adequate fluid (e.g., 0.9% saline solution) is crucial. Examining the product bag and the patient’s identification is mandatory. Meanwhile, vital signs should be closely assessed at 15-min intervals. The lab should be quickly notified for a rapid check of a post-transfusion blood sample. If possible, the bag and tubing should be sent, too. The blood bank is also central in further checking, excluding, or confirming an incompatible transfusion, evaluating the potentially involved antigens [72,73].

## 7. Disseminated Intravascular Coagulation

Disseminated intravascular coagulation (DIC) is a life-threatening acquired pathological process characterized by uncontrolled activation of the coagulation process, leading to hyperfibrinolysis, consumption of platelets and coagulation factors, and finally, to massive hemorrhage or thrombosis. It can be distinguished in two forms in correlation to fibrinolysis: “enhanced fibrinolysis” or a “suppressed fibrinolysis” type, characterized by bleeding or thrombosis, respectively.

It can be triggered by several factors, such as tissue damage (severe tissue injury, burns, stroke, pancreatitis), infections (septic shock, viral infection), vascular disorders (vasculitis, aortic aneurysm), obstetrical complications (abruptio placentae, Hemolysis Elevated Liver enzymes Low Platelet—HELLP—syndrome, eclampsia), liver disease, toxic or immunological reactions (graft versus host disease, snake venom, transplant rejection, severe transfusion reaction) and neoplasms (solid or hematological) [74].

Among the hematological causes, it is mainly associated with acute myeloid leukemia (AML), especially acute promyelocytic (APL) and acute lymphoblastic (ALL), and especially when treated with asparaginase. According to several retrospective studies, the overall incidence of DIC in AML is considered to be 15–25% at diagnosis [75], and it is higher in the presence of hyperleukocytosis, especially in cases of AML that present a normal karyotype with FLT3-ITD mutation [76]. The pathophysiology of DIC and thrombosis in AML is correlated with the production of prothrombotic products such as tissue factor (TF), cancer procoagulant, and cytokines by the blast cells [77]. Additionally, in APL, promyelocyte cells peculiarly produce tissue plasminogen activator (tPA), leading to a higher risk of DIC and, thus, mortality [78]. In ALL, the incidence of DIC is mainly correlated with the administration of asparaginase, as it disrupts the hepatic synthesis of antithrombin, fibrinogen, and other coagulation proteins, increasing the risk of both hemorrhagic and thrombotic events [75].

### 7.1. Clinical Presentation and Diagnosis

The clinical presentation may be quite heterogeneous, as it can be featured with hemorrhagic manifestations, including life-threatening bleeding (cerebral, intra-alveolar hemorrhages), as well as thromboembolic complications, leading to multi-organ dysfunction due to microvascular thrombosis and bleeding. DIC may be divided into two main categories, such as latent and compensated activation of coagulation, in which there is an imbalance between activation and inhibition of the coagulation system. Still, it can be restored by reducing the pro-coagulative stimulus and restoring the inhibitory mechanisms in the coagulation system. In overt DIC, the standard regulatory mechanisms are disrupted, with bleeding and thrombotic manifestations [79]. Modifications in the vessels are also described and are involved in the altered endothelium role [80,81]. Clinical laboratory findings make the diagnosis, and several scoring systems have been proposed to facilitate diagnosis. The International Society on Thrombosis and Haemostasis takes account of clinical symptoms, platelet count, d-dimer, PT increase, and fibrinogen level, with a score ≥5 points compatible with overt DIC [82]. The Japanese Ministry of Health and Welfare (JMHW) proposed a score in which thrombocytopenia would not be considered in patients with hematological malignancies, as it may be connected to bone marrow infiltration [83]. A third score proposed by the Japanese Association for Acute Medicine [84] differs from the other for considering Systemic Inflammatory Response Syndrome (SIRS) parameters (temperature < 36 °C or >38 °C, heart rate > 90 beats/min, respiratory rate > 20 breaths/min or PaCO_2_ < 4.3 kPa, WBC > 12 × 10^9^/L, <4 × 10^9^/L or 10% immature forms present). All scores and their features are summarized in Table 5.

### 7.2. Management

The first aim in treating DIC is to remove the underlying cause, as DIC can be resolved. Supportive therapy is crucial, involving constant monitoring of hemostatic parameters and intensive management of vital functions. The main guidelines for treating DIC report similar features [85,86,87,88]. DIC secondary to AML represents an indication for cytoreduction and chemotherapy. In APL, trans-retinoic acid (ATRA) must be administered as soon as APL is suspected since ATRA has varying effects on the progression of APL and exhibits anticoagulant and antifibrinolytic properties [89]. Transfusion support is fundamental, especially with platelet concentrate (PC) and fresh frozen plasma (PFC), as they can restore low levels of platelets and coagulation factors, particularly fibrinogen. With active bleeding, the platelet transfusion threshold should be considered 50 × 10^9^/L, and fibrinogen replacement therapy is indicated to reach a level >1.5 g/L. A lower threshold of 10 to 20 × 10^9^ platelets/L may be considered in non-bleeding patients who develop DIC after chemotherapy administration. Treatment with anticoagulants may be a rational approach. Still, its use differs among guidelines, and it should be considered when thrombosis is predominant. Low-molecular-weight heparin (LMWH) should be preferred over unfractionated heparin (UFH) [90]. Thrombotic events are more frequently observed in acute lymphoblastic leukemia (ALL), primarily due to the prothrombotic effects associated with the use of L-asparaginase therapy [91], which reduces concentrations of asparagine and hepatic synthesis of antithrombin and fibrinogen. Thus, antithrombin concentrates are recommended by the ISTH to achieve a target level of 80% to 120%, and low-molecular-weight heparin thromboprophylaxis is strongly suggested in patients with a platelet count greater than 30 × 10^9^/L, without bleeding.

## 8. Thrombotic Thrombocytopenic Purpura

### 8.1. Pathophysiology and Symptoms

Thrombotic thrombocytopenic purpura (TTP) is a rare and life-threatening thrombotic microangiopathy characterized by a severe deficiency of A Disintegrin And Metalloprotease with ThromboSpondin type-1 repeats, member 13 (ADAMTS13), a specific von Willebrand factor-cleaving protease, resulting in disseminated microvascular platelet-rich thrombi that cause microangiopathic hemolytic anemia, severe thrombocytopenia, and organ ischemia.

The von Willebrand factor (VWF) is a multimeric glycoprotein with a crucial role in platelet adhesion and aggregation, and the absence of the cleavage factor ADAMTST13 results in the circulation of plasma ultra-large VWF multimers that are hyperadhesive to platelets, causing thrombus formation. ADAMTS13 deficiency is most frequently acquired via ADAMTS13 autoantibodies (immune-mediated TTP, iTTP), but rarely, it is inherited via mutations of the gene encoding ADAMTS13 (congenital TTP) [92].

The first acute episode of TTP usually occurs during adulthood, with a predominant anti-ADAMTS13 autoimmune etiology and a relapsing tendency characterizing its outcome. Rapid recognition of TTP is crucial to start appropriate treatment. Initially, an acute episode of TTP was defined by clinical criteria (multi-visceral ischemic symptoms, mainly neurological) and standard biology criteria (microangiopathic hemolytic anemia and severe thrombocytopenia) occurring without other apparent causes. This definition was recently completed by the evidence of a severe deficiency of ADAMTS13 (activity <10%). Anti-ADAMTS13 IgG may not be detectable in 20% to 25% of acute TTP cases, raising the hypothesis that severe ADAMTS13 deficiency in these patients may result from different, unclear mechanisms [93].

The almost constant signs of TTP are severe thrombocytopenia (typically <30 × 10^9^/L) and microangiopathic hemolytic anemia characterized by schistocytes on the blood smear (at least 1% on the blood smear). Laboratory findings include high reticulocyte count (>120 × 10^9^/L), undetectable serum haptoglobin concentration, elevated lactate dehydrogenase level, increased indirect bilirubin, and negative Coombs test. The clinical onset is often associated with symptoms related to organ ischemia/infarction (i.e., skin and mucosal hemorrhage, weakness, and dyspnea). Most patients have neurologic symptoms at presentation, like headache, confusion and also stroke, coma, and seizures. Ischemia of small vessels can also result in myocardial infarction, mesenteric ischemia, and renal manifestations characterized by isolated proteinuria/hematuria with a serum creatinine level typically below 2 mg/dL. The most frequent clinical conditions associated with TTP are bacterial infections, autoimmune diseases (mainly systemic lupus erythematosus, antiphospholipid syndrome), pregnancy, drugs (mitomycin C, cyclosporine, quinine, clopidogrel, ticlopidine), HIV infection, pancreatitis, cancers, and organ transplantation, with some of them being likely involved in the triggering mechanism of the TTP episode. Recently, a correlation was reported with the SARS-CoV-2 vaccine [94].

The principal differential diagnosis for TTP is hemolytic uremic syndrome (HUS), another thrombotic microangiopathy linked to either Shiga toxin-producing Escherichia coli or abnormalities of proteins of the alternative complement pathway (atypical HUS) [95]. A correct differential diagnosis to distinguish TTP from other thrombotic microangiopathies is crucial because mostly only patients with severe ADAMTS13 deficiency are likely to respond to plasma exchange (PE) [96]. A high troponin value is commonly related to TTP instead of HUS.

### 8.2. Management

The cornerstone of first-line therapy for acute TTP is based on therapeutic plasma exchange supplying the patient’s deficient ADAMTS13, performed daily until hemolysis and features related to organ involvement have resolved and the platelet count has stably recovered. Before starting PE therapy, a blood sample to measure ADAMTS13 activity and inhibitor levels should be drawn. After confirmation of iTTP, with ADAMTS13 <10% in the presence of an inhibitor, targeted treatments should be initiated: continuation of continuous PE; suppression of anti-ADAMTS13 autoantibodies with glucocorticoids and/or other immunosuppressive drugs such as anti-CD20 rituximab; and inhibition of uncontrolled microthrombus formation with caplacizumab. The latter, recently introduced as a standard of care, is a nanobody that targets vWF and inhibits the interaction of all multimers with platelets, with an immediate effect on platelet aggregation and the consequent formation and accumulation of platelet-rich microthrombi [97].

In HERCULES, a pivotal randomized controlled trial, the use of caplacizumab resulted in fewer recurrent iTTP episodes, reduced PE, and a shorter hospital stay. In cases of high suspicion for iTTP, clinicians should consider administering caplacizumab before receiving ADAMTS13 results because the improved benefits of caplacizumab are reported when therapy is started within 3 days of symptom onset [98].

## 9. Sickle Cell Crisis

Sickle cell disease is a hematological disorder caused by a mutation in the hemoglobin β gene, where glutamic acid is replaced by valine on chromosome 11, leading to hemoglobin S (HbS) formation [99]. Blood deoxygenation causes the polymerization of HbS, forming arch-shaped red blood cells called “sickle cells”. Different conditions, such as hypoxia, obstructive sleep apnea, acidosis, fever, infections, and dehydration, cause deoxygenation. These abnormal red blood cells tend to break into small vessels, causing hemolytic anemia and vascular occlusion, resulting in organ ischemia and damage [100]. Recurrent vaso-occlusions lead to debilitating chronic arthritis due to osteonecrosis in the joints, progressive retinopathy, chronic kidney failure, a higher risk of stroke, and a reduced lifespan [100].

The only curative options are hematopoietic stem cell transplant (HSCT) and gene editing. Using either myeloablative or reduced-intensity conditioning, HSCT has a 5-year overall survival rate of around 90% in cases of HLA-identical donors. Conversely, matched unrelated transplants face challenges, including a 29% incidence of graft-versus-host disease (GVHD) [101]. Gene therapy is emerging as a treatment option, where functional human beta-globin genes (e.g., lentiglobin) are introduced ex vivo into the patient’s stem cells, producing HbA and significant reductions in hemolysis and vaso-occlusive crises [102]. Cell-based gene therapy using CRISPR/Cas9 targets the defective HbB gene, aiming to correct the genome. Studies still need to validate the long-term effectiveness and safety of CRISPR/Cas9 [103].

### Diagnosis, Features, and Management

The term “sickle cell crisis” is used to describe several acute conditions such as the vaso-occlusive crisis (VOC), acute chest syndrome, splenic sequestration crisis, hyperhemolytic crisis, hepatic crisis, dactylitis, and aplastic crisis. It also includes pneumonia, meningitis, sepsis and osteomyelitis, stroke, avascular necrosis, priapism, and venous thromboembolism [104]. Approximately one-third of deaths related to sickle cell disease occur within 24 h of the insurgence. Typically, adults are more likely to report chronic complications, while children tend to experience acute fatalities [100].

VOC represents a hematological emergency characterized by episodes of excruciating pain that can develop in life-threatening events [105]. In some cases, these events are manageable at home; in others, they require hospitalization [106]. Biomarkers help recognize VOC quickly, allowing therapy onset and reducing complications. They also aid in identifying risk categories of patients and guiding treatment [100]. Advanced age, high hemoglobin levels or high hematocrit, α-thalassemia, and low fetal hemoglobin seem to be related to a higher frequency of VOC episodes [107].

Effective treatment of VOC requires early diagnosis, prevention of complications, and management of organ damage. Prompt pain assessment and analgesia initiation are essential. Depending on severity, analgesics can be administered intravenously, intranasally, or orally for less severe cases. Individualization of type, route, and dose is crucial, with guidelines recommending early parenteral opioids, typically morphine at 0.1 mg/kg intravenous or subcutaneous every 20 min, followed by maintenance doses of 0.05 to 0.1 mg/kg every 2 to 4 h, with close monitoring of vital signs. Patients with persistent pain may benefit from a patient-controlled analgesia pump [104]. If pain remains uncontrolled, hospitalization and more potent analgesics or transfusions may be needed [104]. Acetaminophen (paracetamol) and non-steroidal anti-inflammatory drugs (NSAIDs) are commonly used alongside opioids as adjunct therapies. They not only help lower the need for opioids and minimize related side effects, but they also lower the increased inflammatory markers, such as prostaglandins, associated with VOC [100]. Possible concomitant treatments include hydration, laxatives, antihistamines, antiemetics, intravenous fluids, and oxygen for hypoxic patients. Other approaches should be applied in chronic conditions, such as anxiolytics or non-pharmacological strategies like massage, acupuncture, yoga, and meditation [107,108].

As mentioned before, added to VOC, chest syndrome and splenic sequestration require supportive care, including hydration, oxygen administration, and blood transfusion, as well as avoiding excessive sedation with close monitoring of oxygen saturation and respiratory status. Empiric antibiotics, adequate analgesia, and simple or exchange transfusions may be necessary for acute chest syndrome. Splenic sequestration crisis with hypovolemic shock requires aggressive treatment, as inadequate management increases mortality risk [102]. 

Other conditions are possible complications during the crisis [104]. “Hemolytic crisis” is defined by a sudden drop in hemoglobin level, increased LDH, bilirubin, decreased reticulocyte count, and dark-colored urine. A specific form of hemolytic crisis, hyperhemolysis syndrome, is a complication of repeated blood transfusions in which lysis of both native and donor erythrocytes occurs. Clinicians must discontinue the blood transfusion and consider intravenous immunoglobulin with steroids as premedication. Strokes are another complication of sickle cell crisis, which involves penetrating arteries and leads to cognitive defects. Treatment consists of red cell exchange for acute strokes and keeping HbS below 30% with transfusions for chronic strokes. In this case, HSCT is a strict indication of sickle cell disease.

Priapism is an unwanted penile erection caused by the swelling of the tunica albuginea secondary to vascular dysregulation. This condition, possible in sickle cell crisis, can occur for more than four hours; shunt surgery or corporeal aspiration is needed. 

Girdle syndrome is a rare condition characterized by pain that resembles a girdle, caused by vaso-occlusion in the lungs, liver, and mesentery. An abdominal-pelvic CT scan with contrast could underline bowel wall thickening, mucosal hyperenhancement, and submucosal edema.

The infrequent occurrence of ischemic bowel may result from the rich collateral circulation in the mesentery and bowel wall, providing a protective effect. In uncomplicated cases, supportive care generally leads to resolution, while severe cases may result in bowel perforation, necessitating emergency surgery.

Hydroxyurea is a potent inducer of fetal hemoglobin (HbF), which is protective in sickle cell patients. It helps prevent VOCs, reduce hospitalization, morbidity, and mortality, and is used carefully with blood count monitoring for the well-known risk of myelosuppression [109].

## 10. Cytokine Release and Immune Effector Cell-Associated Neurotoxicity Syndromes

Chimeric antigen receptor T-cell (CAR-T) therapy significantly advances hematologic oncology, harnessing the patient’s immune system to recognize and attack malignant cells. CAR-T therapy involves genetically modifying T cells to express specific receptors that recognize cancer cells, leading to their targeted destruction [110]. The same effects are described for bispecific antibodies, widely used nowadays in multiple myeloma, ALL or lymphomas [111,112,113]. Chief among these toxicities are cytokine release syndrome (CRS) and immune effector cell-associated neurotoxicity syndrome (ICANS), both of which are recognized as medical emergencies in hematology due to their potentially life-threatening consequences. Early diagnosis and intervention are paramount in managing these toxicities effectively to improve patient outcomes.

CRS is an acute inflammatory response triggered by the massive release of cytokines from CAR-T cells and the patient’s immune cells, in response to tumor cell lysis. The activation of CAR-T cells leads to a cascade effect, whereby cytokines such as interleukin-6 (IL-6), interferon-gamma (IFN-γ), and TNF-α are released, driving widespread immune activation. While this inflammatory surge is critical for the anti-tumor effects of CAR-T therapy, it can also lead to significant systemic toxicity, characterized by a hyperinflammatory state affecting multiple organ systems [114]. ICANS, conversely, is a severe CAR-T-related toxicity that primarily affects the central nervous system (CNS). While the precise mechanism underlying ICANS remains unclear, it is thought to involve an inflammatory response within the CNS, exacerbated by elevated levels of cytokines like IL-1, IL-6, and IFN-γ. Disrupting the blood–brain barrier due to these cytokines may allow immune cells and inflammatory mediators to infiltrate the CNS, resulting in neurotoxicity. ICANS can occur independently of CRS but often coexists, particularly in severe cases of CRS [115,116].

### 10.1. Classification, Incidence, and Diagnosis

Grading scales, such as the American Society for Transplantation and Cellular Therapy (ASTCT) consensus grading system, stratify the CRS/ICANS into four degrees, considering the presence of fever (< or ≥38 °C), hypotension (requiring vasopressin treatment or not), and hypoxia (requiring oxygen or CPAP/intubation) [117].

The incidence of CRS varies depending on the specific CAR-T product, tumor type, and patient factors. Approximately 70–90% of patients receiving CAR-T therapy, particularly those treated with CD19-targeted CAR-T cells, experience some degree of CRS. Around 15–30% of these cases may develop severe CRS (grade 3 or higher). Patients with higher tumor burdens or those receiving high doses of CAR-T cells are at increased risk, as more malignant cells can prompt an amplified cytokine response. It typically presents within the first few days following CAR-T cell infusion, with symptoms peaking between days 1 and 14. Laboratory markers, particularly elevated levels of C-reactive protein (CRP), ferritin, and IL-6, can aid in diagnosis but are not definitive without corresponding clinical symptoms.

Conversely, the incidence of ICANS also varies depending on the CAR-T product and patient population, with reports indicating an occurrence rate of around 30–50% among patients treated with CD19-targeted CAR-T cells.

Severe ICANS (grade 3 or higher) is less common, occurring in approximately 10–15% of cases. Certain factors, such as preexisting neurological conditions, high CAR-T cell doses, and prior episodes of severe CRS, may increase the risk of developing ICANS. The incidence of ICANS also varies depending on the CAR-T product and patient population, with reports indicating an occurrence rate of around 30–50% among patients treated with CD19-targeted CAR-T cells [118]. 

ICANS typically develops several days to a few weeks post-CAR-T infusion, with a peak incidence of around 5–10 days. Symptoms can range from mild cognitive impairment and headache to more severe manifestations such as seizures, aphasia, and cerebral edema [119]. The ASTCT provides a standardized grading system for ICANS based on the Immune Effector Cell-Associated Encephalopathy (ICE) score, which evaluates cognitive function, orientation, language abilities, and other neurological functions [120].

Diagnostic workup may include electroencephalography (EEG) to monitor for seizures, brain imaging to detect cerebral edema, and lumbar puncture to assess cerebrospinal fluid (CSF) inflammatory markers. Elevated serum and CSF cytokine levels, particularly of IL-6, are often associated with ICANS, though they are not specific markers.

Signs and symptoms are reported in Table 6.

### 10.2. Management

Timely intervention is critical in managing CRS and ICANS. The mainstay of treatment includes immunosuppressive agents such as corticosteroids and cytokine inhibitors. Tocilizumab, an IL-6 receptor antagonist, is FDA-approved for managing CRS and has shown effectiveness in mitigating symptoms by dampening the IL-6-mediated inflammatory response. For severe cases, mainly those unresponsive to tocilizumab, corticosteroids such as dexamethasone are administered to suppress the overall immune response [121]. In cases of refractory CRS, additional agents such as siltuximab (an IL-6 ligand inhibitor), anakinra (an IL-1 receptor antagonist), or ruxolitinib (Janus kinase inhibitor commonly used in myeloproliferative neoplasms and GVHD) may be considered [122,123,124,125,126,127]. Management also includes supportive care, including vasopressors for hypotension and supplemental oxygen or mechanical ventilation for respiratory support. In addition, corticosteroids, particularly dexamethasone, are the cornerstone of ICANS management, as they can cross the blood–brain barrier and effectively reduce CNS inflammation [121]. Additionally, prophylactic anti-epileptics, such as levetiracetam, are commonly used to prevent seizure activity in high-risk patients. In severe cases, osmotic agents like mannitol may be administered to reduce cerebral edema [124].

A brief representation for the management of CRS is reported in Table 7.

## 11. Other Emergencies

Adrenal insufficiency (AI) is a potentially life-threatening condition that can complicate hematological disorders. It often arises due to infiltration of the adrenal glands by hematologic malignancies such as lymphoma or leukemia, or as a consequence of treatment regimens including chemotherapy, radiation, or immunosuppressive therapy [128]. AI may also develop in the context of hemophagocytic lymphohistiocytosis (HLH) or as a paraneoplastic syndrome. Symptoms of AI in these settings can be nonspecific, such as fatigue, hypotension, and electrolyte imbalances, complicating the potential diagnosis [129]. Hematological conditions associated with disseminated intravascular coagulation or massive hemorrhage can lead to adrenal hemorrhage, further contributing to adrenal dysfunction. Early recognition and management of AI, often with glucocorticoid replacement therapy, are critical to improving outcomes and mitigating the risk of adrenal crisis [130]. On the other hand, cardiovascular emergencies in hematological conditions represent critical scenarios that require prompt recognition and management. For instance, severe anemia can precipitate high-output heart failure, while hyperviscosity syndromes, seen in conditions such as MPNs where the thrombotic risk is increased, and prothrombotic states associated with malignancies or chemotherapy can lead to reduced perfusion and ischemic events [131,132].

Rapid assessment, stabilization, and targeted therapies are essential to manage these life-threatening conditions effectively and improve outcomes for patients with hematological disorders.

## 12. Conclusions

Emergencies in hematology require rapid recognition and targeted management to prevent severe morbidity and mortality. Effective intervention, guided by standardized protocols and multidisciplinary teams, is essential to improve outcomes and support patients, ensuring a higher survival rate.

## Figures and Tables

**Table 1:** Therapeutic approaches for hypercalcemia. Revised from Gupta, S et al., Cureus 2023 [6].

Grade of Hypercalcemia	Approach
Mild hypercalcemia (Ca^2+^ < 12 mg/dL)	Does not require aggressive treatment Avoid loop diuretics, lithium carbonate, volume depletion, prolonged inactivity, high-calcium diet (>1000 mg/day), and calcium/vitamin D supplements since they could worsen hypercalcemia Adequate hydration (6–8 glasses of water per day)
Moderate hypercalcemia (12–13.9 mg/dL)	Intravenous saline hydration (0.9%) at twice the maintenance rate until any fluid deficit is replaced and diuresis is achieved (≥200–300 mL/h) Bisphosphonate, e.g., disodium pamidronate 60–90 mg IV over 2 h, or zoledronic acid 4 mg IV in 100 mL normal saline over at least 15 min Hemodialysis for patients with heart failure or renal insufficiency
Severe hypercalcemia (>14 mg/dL)	Simultaneous administration of intravenous isotonic saline (4–6 L/24 h) Subcutaneous calcitonin (200 IU every 8 h), which guarantees a mild and time-limited effect Bisphosphonate, e.g., disodium pamidronate 60–90 mg intravenous over 2 h, or zoledronic acid 4 mg IV in 100 mL normal saline over at least 15 min Other treatments (gallium nitrate, denosumab, cincalcet, glucocorticoid therapy) Hemodialysis for patients with heart failure or renal insufficiency

**Table 2:** Leukostasis grading score, as reported in the text, adapted from Novotny, JR et al., Eur J Haematol 2005 [26].

Grade	Probability of Leukostasis Syndrome	Symptoms
0	Not present	No respiratory or neurological symptoms
1	Low	Mild symptoms, moderate fatigue, headache, dizziness
2	Intermediate	Marked fatigue, shortness of breath, vision impairment, marked headache, tinnitus
3	High	Dyspnea, acute distress respiratory syndrome, focal neurological signs, coma or symptoms related to microvascular occlusion

**Table 3:** Leukostasis-related clinical manifestations. CNS: central nervous system; GI: gastrointestinal; ARDS: acute respiratory distress syndrome.

Grade	Symptoms	Signs
CNS	Confusion, lethargy, tinnitus, headache	Intracranial hemorrhage and hypertension
Lungs	Shortness of breath, cough, tachypnea, dyspnea	Acute pulmonary edema, ARDS
Heart	Chest pain, peripheral ischemia, ECG abnormalities	Congestive heart failure, infarction
Eyes	Blurred vision, diplopia, hemianopia	Papilledema, retinal hemorrhage
GI tract	Diarrhea, anorexia, nausea, and vomiting	Acute appendicitis
Spleen	Abdominal pain, fever, hypotension	Rupture of spleen
Kidney		Acute kidney injury, renal vein thrombosis,
Vessels		deep venous thrombosis, bowel ischemia, priapism, avascular necrosis of the femoral head

**Table 4:** Acute and delayed transfusion reactions, divided into two categories depending on the time of onset from transfusion initiation.

Acute Transfusion Reactions	Delayed Transfusion Reactions
Acute hemolytic reactions	Delayed hemolytic reactions
Febrile nonhemolytic transfusion reactions	Post-transfusion purpura
Allergic transfusion reactions	
Transfusion-related acute lung injury	
Transfusion-associated circulatory overload	
Transfusion-related sepsis	

**Table 5:** Scores for DIC diagnosis. ISTH: International Society on Thrombosis and Haemostasis; JMHV: Japanese Ministry of Health and Welfare; JAAM: Japanese Association for Acute Medicine; FDPS: fibrin degradation products; NC: not considered.

	ISTH	JMHV	JAAM	SCORE
Clinical symptoms	NC	Organ failure	NC	1
SIRS criteria	NC	NC	0–2 criteria	1
			≥3 criteria	1
Platelet count	>100 × 109/L	NC	≥120 × 109/L	0
	50–100 × 109/L		80–120 × 109/L	1
	<50 × 109/L		/	2
			<80 × 109/L	3
D-dimers or FDPS	No increase	FDPS (≥)20	≤10 µg/mL	0
	Moderate increase		10–25 µg/mL	1
	Severe increase		/	2
			≥25 µg/mL	3
PT increase or PT ratio	<3 s	1.25–1.67	<1.2	0
	3–6 s	>1.67	≥1.2	1
	>6 s			2
Fibrinogen	≥1 g/L	NC	>1.5 g/L	0
	<1 g/L		1.0–≤1.5 g/L	1
			<1.0 g/L	2
**Diagnosis of DIC**	≥5 points	≥4 points	≥4 points	

**Table 6:** Clinical features of CRS and ICANS, according to ASTCT consensus grading system [117]. Temp: temperature; NC: nasal cannula; O_2_: supplemental oxygen; HFNC: high-flow nasal cannula (>6 L/min); NA: not applicable; CN: cranial nerve; CRS: cytokine release syndrome; ICANS: immune effector cell-associated neurotoxicity syndrome.

Toxicity	Parameter	Grade 1	Grade 2	Grade 3	Grade 4
CRS	Fever	Temp ≥ 38 °C	Temp ≥ 38 °C	Temp ≥ 38 °C	Temp ≥ 38 °C
	Hypotension	None	No pressor requirement	Pressor requirement with or without vasopressin	Multiple pressors excluding vasopressin
	None	O_2_ by low-flow NC (≤6 L/min) or blow-by	O_2_ by HFNC, facemask, nonrebreather mask or Venturi mask	Positive pressure ventilatory support *	None
ICANS	ICE SCORE †	7–9	3–6	0–2	0
	Depressed consciousness	Awakens spontaneously	Awakens to voice	Awakens only to tactile stimulus	Arousable with vigorous tactile stimuli, unarousable, stupor, or coma
	Seizures	NA	NA	Any seizure with rapid clinical resolution	Prolonged (>5 min), non-self resolving
	Motor findings	NA	NA	NA	Significant focal motor weakness
	Elevated ICP/ cerebral edema ‡	NA	NA	Focal edema on brain imaging	Diffuse edema on imaging, or decerebrate/decorticate posturing, CN VI palsy, or Cushing’s triad

Notes: * Positive pressure ventilation includes continuous positive airway pressure (CPAP), bilevel positive airway pressure (BiPAP), or mechanical ventilation. † Immune Effector Cell-Associated Encephalopathy (ICE) score includes points for orientation (year, month, city, hospital; 4 points), naming (three objects; 3 points), following simple commands (1 point), writing a simple sentence (1 point), and attention (e.g., counting backwards from 100 by 10; 1 point). ‡ Excludes intracranial hemorrhage or associated edema.

**Table 7:** Management of CRS. SBP: systolic blood pressure; ICU: intensive care unit; IV: intravenous.

CRS Grade	Management
GRADE 1	Supportive care including analgesics and antipyretics. If fever, treat for neutropenic infections protocol. Consider tocilizumab for persistent (lasting > 3 days) and refractory fever
GRADE 2	IV fluid bolus 500–1000 mL to maintain SBP > 90 mmHg. Administer tocilizumab early if persistent fever of ≥39 °C, hypotension after initial fluid bolus, or initiation of oxygen supplementation If persistent hypotension after two fluid bolus and tocilizumab, transfer to ICU for consideration of low-dose vasopressor therapy Add dexamethasone 10 mg IV 6 hourly if hypotension persists after anti-IL-6 therapy, high risk for severe CRS, worsening hypoxia, or clinical concern
GRADE 3	Intensive care should be considered Administer tocilizumab Add steroids if unresponsive within 24 h dexamethasone 10 mg IV every 6 h If refractory, increase to 20 mg IV every 6 h If unresponsive CRS, add anakinra Consider anti-tumor necrosis factor (TNF) antibodies as clinically appropriate. Perform echocardiogram if persistent hypotension
GRADE 4	Intensive care. Administer tocilizumab High-dose methylprednisolone 1 g/day IV If unresponsive CRS, add anakinra If unresponsive, consider alternative agents such as anti-TNF and other agents
GRADE 5	Death
